# A Cisplatin-Based Prodrug Inhibits Nucleotide Excision Repair Independently of Chromatin Accessibility to Overcome Resistance

**DOI:** 10.3390/biom16040542

**Published:** 2026-04-07

**Authors:** Ya’ara Negev-Korem, Hadar Golan-Berman, Elisheva Heilbrun, Subhendu Karmakar, Yoram Soroka, Marina Frušić-Zlotkin, Ofer Chen, Hiba Hassanain, Esther Stern, Ori Wald, Dan Gibson, Ron Kohen, Sheera Adar

**Affiliations:** 1 Department of Microbiology and Molecular Genetics, The Institute for Medical Research Israel-Canada, The Faculty of Medicine, The Hebrew University of Jerusalem, Jerusalem 9112102, Israel; yaara.negev@mail.huji.ac.il (Y.N.-K.); hadar.golan@mail.huji.ac.il (H.G.-B.); elisheva.heilbrun@mail.huji.ac.il (E.H.); ofer.chen2@mail.huji.ac.il (O.C.); hiba.hassanain@mail.huji.ac.il (H.H.); 2 The Myers Skin Research Laboratory, Institute for Drug Research, School of Pharmacy, The Faculty of Medicine, The Hebrew University of Jerusalem, Jerusalem 9103401, Israel; yorams@ekmd.huji.ac.il (Y.S.); marina.zlotkinfrusic@mail.huji.ac.il (M.F.-Z.); ronk@ekmd.huji.ac.il (R.K.); 3 Institute for Drug Research, School of Pharmacy, The Faculty of Medicine, The Hebrew University of Jerusalem, Jerusalem 9112102, Israel; sksubhenduk@gmail.com (S.K.); dang@ekmd.huji.ac.il (D.G.); 4 Department of Chemistry, Sister Nivedita University, New Town, Kolkata 700156, West Bengal, India; 5 Gene Therapy Institute, Hadassah Medical Center, The Faculty of Medicine, The Hebrew University of Jerusalem, Jerusalem 9112102, Israel; ester.stern@mail.huji.ac.il (E.S.); ori.wald@mail.huji.ac.il (O.W.); 6 Department of Cardiothoracic Surgery, Hadassah Medical Center, The Faculty of Medicine, The Hebrew University of Jerusalem, Jerusalem 9112001, Israel

**Keywords:** cisplatin, DNA damage, DNA repair, nucleotide excision repair, chromatin, KDAC, Nrf2, Pt(IV)

## Abstract

Cisplatin [*cis*-diamminedichloroplatinum(II)] is a widely used chemotherapeutic agent that induces cytotoxicity primarily through DNA damage; however, drug resistance severely limits its efficacy. Cisplatin resistance is complex and multifactorial, involving DNA repair via nucleotide excision repair (NER), increased detoxification activities, and overexpression of lysine deacetylases (KDACs), which reduce chromatin accessibility and alter transcriptional regulation. Combining cisplatin with KDAC inhibitors has shown promise, often attributed to increased drug sensitivity through higher chromatin accessibility; however, this hypothesis has not been validated. Here, we synthesized a novel Pt(IV) derivative, *ctc*-[Pt(NH_3_)_2_(VPA)(PhB)Cl_2_] (cPVP), which combines cisplatin with two KDAC inhibitors, phenylbutyrate and valproic acid. Compared with cisplatin, cPVP induced significantly greater cytotoxicity, and increased DNA damage formation. High-resolution mapping of genomic cisplatin damage and repair indicated that enhanced sensitivity resulted not from altered chromatin accessibility, but from increased drug uptake and the inhibition of NER. Moreover, cPVP prevented the development of resistance to both cisplatin and itself in cancer cells. Together, these results establish the inhibition of nucleotide excision repair, rather than enhanced damage sensitivity due to chromatin accessibility, as the primary mechanism by which KDAC-targeting cisplatin prodrugs overcome resistance to platinum-based therapies.

## 1. Introduction

Since its approval in 1979, the metallo-drug cisplatin [cis-diamminedichloroplatinum(II)] is widely used as a chemotherapeutic agent [1,2] to treat multiple tumor types, notably testicular and ovarian cancers [3,4], but also lung cancer, head and neck cancer, bladder cancer, cervical cancer and others [5,6]. The continued use of cisplatin is greatly restricted by severe dose-limiting side effects due to a lack of tumor selectivity [7,8,9], and intrinsic or acquired drug resistance limiting cisplatin efficacy [9,10,11].

Cisplatin cytotoxicity is mainly attributed to its covalent intrastrand interaction with DNA to form DNA adducts [12,13]. The main mechanism for eliminating cisplatin DNA adducts in human cells is nucleotide excision repair (NER) [14,15]. NER is divided into two sub-pathways that differ in the damage recognition step: global genome repair (GGR), in which repair factors recognize damage directly throughout the genome, and transcription-coupled repair (TCR), in which an RNA polymerase that is blocked by the damage acts as the damage recognition factor, recruiting repair factors to actively transcribed regions. After damage recognition, the two pathways share the subsequent steps of repair, which involve the excision of a single strand oligonucleotide containing the lesion (~26 nt) from the genome, followed by gap filling by DNA polymerase and ligation by a DNA ligase [16,17,18,19].

Increased NER has been implicated in cisplatin resistance [20,21], which is complex and multifactorial as it involves numerous cellular pathways and activities [9,10,11,22]. The accelerated rate of adduct repair may attenuate the apoptotic process and thus facilitate the survival of resistant cells [14,20].

NER efficiency is modulated by the packaging of DNA into nucleosomes and higher-order chromatin [19,23]. High-resolution measurements of damage and repair across the genome have shown that accessible genomic regions are repaired with higher efficiency [24,25,26]. Lysine deacetylases (KDACs, also termed histone deacetylases, HDACs), are key epigenetic modifiers [22,27] which remove acetyl groups from lysine residues within histone tails, leading to chromatin condensation. KDACs thus limit access to DNA strands and promote transcriptional repression [28,29]. Additionally, these enzymes are known to deacetylate lysine residues of non-histone proteins, thus regulating numerous biological processes and widely affecting cellular functioning [30]. Aberrant activity of KDACs is known to promote resistance to DNA-targeting agents, including cisplatin, by a mechanism that is not entirely clear [31].

**Figure 1:**
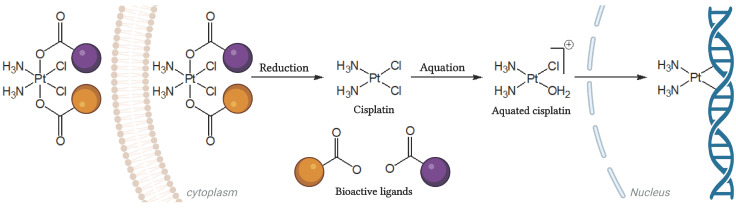
Pt(IV) prodrug. A triple-component Pt(IV) prodrug of cisplatin axially modified by two different bioactive carboxylate ligands (orange and purple). It is inert outside the cell and is activated intracellularly by reduction to the origin moieties. This is followed by aquation of cisplatin to attain DNA covalent binding.

Pt(IV) derivatives of cisplatin have been developed precisely for the purpose of combining cisplatin treatment with additional anti-cancer drugs. These multi-targeting prodrugs can release cisplatin and additional bioactive moieties inside the cancer cells, thereby enhancing the chances of overcoming resistance and increasing the therapeutic index. The conversion of four coordinate Pt(II) drugs (e.g., cisplatin) to six coordinate Pt(IV) derivatives involves their oxidation followed by axial modification with bioactive ligands, to form multi-action prodrugs [32,33]. As the original cisplatin, these molecules are likely to also harm normal dividing cells more efficiently. However, these compounds are stable in culture medium and are considered relatively inert and stable outside tumor cells, hence they are expected to have an increased bioavailability and reduced side effects compared to the original Pt(II) drug [33,34,35]. When entering the cell, Pt(IV) prodrugs are reduced and release the Pt(II) parent drug and the bioactive molecules, enabling interactions with their intracellular targets (Figure 1) [36]. Accordingly, Pt(IV) prodrugs have the potential to evade cisplatin resistance and present improved pharmacology, as the bioactive moieties have the potential to act synergistically and efficiently to eradicate cancer cells [33,34].

In this study, we have selected 4-phenylbutyric acid (PhB) and valproic acid (VPA) as the bioactive ligands to be conjugated with cisplatin. Both PhB and VPA are KDAC inhibitors (KDAC*i*), thus enabling us to explore the contribution of chromatin accessibility to cisplatin treatment [37,38]. These specific inhibitors were chosen because they possess carboxylic groups, which facilitate conjugation with Pt(IV) [32]. Upon reduction, the bond between Pt(IV) and the carboxylate oxygen is broken, releasing the inhibitors in their active form [36]. Previously reported dual-component Pt(IV) derivatives of cisplatin with either two PhB or two VPA axial ligands have demonstrated anti-cancer activity and significantly improved cytotoxicity compared to cisplatin [39]. However, the combination of both PhB and VPA in a single agent was not reported. Here, we synthesized a novel triple-component Pt(IV) prodrug consisting of PhB and VPA fused to cisplatin, and tested its effect on cisplatin damage formation, repair, cytotoxicity and resistance. We conducted in-depth genomic characterization of the damage induced by this prodrug treatment and its effect on chromatin accessibility and DNA repair capacity. Our results indicate KDAC inhibition increases damage sensitivity in cells, and cPVP holds the potential to overcome cisplatin resistance.

## 2. Materials and Methods

### 2.1. cPVP Synthesis

The procedure employed here followed the synthetic procedures of the triple-action Pt(IV) derivatives described in the literature [40]. A solution of valproic anhydride (75 mg, 0.28 mmol) dissolved in 1 mL of DMSO (Bio-Lab Ltd., Jerusalem, Israel) was added to a suspension of oxoplatin (60 mg, 0.18 mmol) in DMSO (5 mL) and stirred over-night at room temperature. After confirming the formation of monocarboxylato monohydroxido Pt(IV) by ^195^Pt NMR chemical shift (1037.1 ppm), the clear yellow reaction mixture was extracted several times with a mixture of diethyl ether and petroleum ether (20:1; Bio-Lab Ltd., Jerusalem, Israel) to achieve a yellow sticky solid. It was then lyophilized over-night to remove the remaining solvent traces and used in the next step without further purification. The crude solid (71 mg, 0.15 mmol) was mixed with 4-phenylbutyric anhydride (235 mg, 0.76 mmol) following dissolution in 5 mL of acetonitrile (HPLC grade; J.T. Baker, Phillipsburg, NJ, USA). After stirring over-night at room temperature, the reaction mixture was injected directly in preparative RP-HPLC (UltimaMate 3000 station, Thermo Scientific, Waltham, MA, USA) equipped with a reverse-phase C18 column (Phenomenex Luna: length 250 mm, internal diameter 21.2 mm, particle size 10 μm, pore size 100 Å) and purified with (0–100)% acetonitrile gradient ran over 15 min followed by 15 min at 100% acetonitrile. The pure fractions were combined before lyophilization to afford the desired product as bright yellow flaky solid. Yield 17 mg (20%). RT = 13.5 min (purity > 97%). ^195^Pt NMR (107.5 MHz, MeOD) δ: 1102; ^1^H NMR (500 MHz, MeOD) δ: 7.27–7.18 (m, 4H, ArH_PhB_), 7.15 (m, 1H, ArH_PhB_), 6.58 (br, NH_3_), 2.66 (t, 2H, J = 7.6 Hz, ^γ^CH_2 PhB_), 2.43–2.34 (m, 3H, ^α^CH_2 PhB and_ CH_Val_), 1.93–1.85 (m, 2H, ^β^CH_2 PhB_), 1.61–1.53 (m, 2H, CH_2 Val_), 1.40–1.29 (m, 6H, CH_2 Val_), 0.91 (t, 6H, J = 7.1 Hz, CH_3 Val_); ^13^C NMR (125 MHz, MeOD) δ: 187.4, 184.1, 143.4, 129.7, 129.3, 126.7, 48.6, 36.4, 36.2, 28.9, 21.7, 14.5; ESI-MS calculated for [C_18_H_32_Cl_2_N_2_O_4_Pt + Na]^+^: 628.13, found 629.06; elemental analysis (%) calculated for C_18_H_32_Cl_2_N_2_O_4_Pt. 2.5H_2_O: C 33.18, H 5.72, N 4.30, found C 32.99, H 5.04, N 4.65. For anhydrides synthesis, 50 mM solution of starting free acid in chloroform was stirred over-night in the presence of 0.6 Eq of EDC. The mixture was washed three times with citric acid aqueous solution (1 g/100 mL), and three times with sodiumbicarbonate aqueous solution (1 g/100 mL). It was then dried on sodium sulphate, and finally evaporated to dryness under reduced pressure.

### 2.2. Cell Culture

A2780 cells (93112519, Sigma-Aldrich, Rehovot, Israel) were cultured in RPMI 1640 Medium (Biological Industries (BI), Beit Haemek, Israel), supplemented with 10% (*v*/*v*) fetal bovine serum (FBS; BI), 2 mM L-glutamine (BI), 1 mM sodium pyruvate (BI), 100 U/mL penicillin and 0.1 mg/mL streptomycin (BI). A549 cells (CCL-185^TM^, ATCC, Manassas, VA, USA) were cultured in DMEM (BI) containing 4.5 g/L D-glucose and supplemented with 10% (*v*/*v*) FBS, 2 mM L-glutamine, 1 mM sodium pyruvate, 100 U/mL penicillin and 0.1 mg/mL streptomycin.

All cultures were maintained in a humidified 5% CO_2_ incubator at 37 °C. Cells were sub-cultured every 3–4 days to maintain logarithmic growth. Cell seeding for all experiments was done 24 h before treatments, allowing cell adherence.

### 2.3. Cytotoxicity

The cytotoxic effects of cisplatin (Pharmachemie BV, Teva group, Haarlem, The Netherlands) and cPVP against A2780 and A549 tumor cells were evaluated using the well-established MTT [3-(4,5-dimethylthiazol-2-yl)-2,5-diphenyltetrazolium bromide] colorimetric assay [41]. In short, cells were seeded into a 96-well plate at a density of 5 × 10^3^, 2.5 × 10^3^, and 7 × 10^3^, respectively, before treatment with ascending concentrations (0.0–20,000.0 nM) of cisplatin or cPVP for 72 h. Media was then replaced with 0.5 mg/mL MTT solution (Sigma-Aldrich, Rehovot, Israel) for one-hour incubation, which was finally replaced with 100 µL/well DMSO. Absorbance was recorded at λ = 570 nm and 690 nm, using Cytation 3 Imaging Reader (BioTek, Winooski, VT, USA). Final results were obtained from Δ OD (570–690) nm. IC_50_ values were calculated using the online calculator from AAT Bioquest [42].

### 2.4. DNA Sample Preparation for Inductively Coupled Plasma Mass Spectrometry (ICP-MS) Analysis

For DNA damage evaluation, A2780 cells were seeded at 2 × 10^6^ cells/60 mm dish and A549 at 2 × 10^6^ cells/100 mm dish. Cells were incubated with 3–40 µM cisplatin or cPVP for two hours and then harvested for initial damage levels. For repair assay, the drug-containing media was replaced with fresh media for repair–incubation of 24–48 h. To extract DNA for damage analysis, cells were harvested by scraping into ice-cold PBS (BI).

For the assessment of DNA damage accumulation post-treatment, A2780 cells were seeded at a density of 2 × 10^6^ cells/60 mm dish. Cells were treated with 3 µM of cPVP or 20 µM of cisplatin for two hours, the drug containing media was removed, cells were washed with PBS, and were then incubated in fresh media for 1–4 h. At each time point cells were harvested by scraping into ice-cold PBS.

In all cases DNA was extracted by using DNeasy^®^ Blood & Tissue kit (69504, Qiagen, Hilden, Germany), and quantified by the QuantiFluor^®^ dsDNA System (E2670, Promega corporation, Madison, WI, USA), following manufacturers’ protocols.

### 2.5. Cells Samples Preparation for ICP-MS Analysis

To assess cellular accumulation of platinum, A2780 cells were seeded in 6-well plates, at a density of 1 × 10^6^ cells/well. After the indicated treatments, cells were washed once with PBS and harvested by scrapping into 0.4 mL of fresh PBS.

### 2.6. ^195^Pt Content Determination by ICP-MS

Prior to analysis, DNA or cell samples were subjected to acidic digestion. DNA (2 µg) or cell suspension in PBS (0.1 mL) were transferred to scintillation bottles with Silicon/Teflon caps (CSI analytical innovations, Petach-Tikva, Israel) and 1 mL of 70% nitric acid (HNO_3_; Bio-Lab Ltd., Jerusalem, Israel) was added. Properly capped samples were placed in a sand-bath and heated for two hours at 90 °C. Then, caps were removed and full evaporation was allowed over-night at the same heat. Samples were re-suspended in 1 mL of 1% HNO_3_, followed by vigorous vortex and shaking. Samples were centrifuged at 10,000 rpm for 10 min at room temperature and transferred to ICP-MS-suitable polypropylene scintillation vials (Thermo Fisher Scientific Inc., Waltham, MA, USA). Calibration curve ranging from 0 to 10,240 pg/mL was freshly prepared from the commercial cisplatin solution (1 mg/mL) diluted with HPLC-grade water (J.T. Baker, Phillipsburg, NJ, USA), and was subjected to the same procedure described. Samples and calibration curve were measured by Agilent 8900 Triple Quadrupole ICP-MS instrument at the Institute of Earth Sciences core facility of The Hebrew University of Jerusalem. ^195^Pt content of DNA samples was normalized to µg of DNA for comparison.

### 2.7. Damage-Seq

A2780 cells were seeded at 5 × 10^6^ cells per 100 mm dish before incubation with 200 µM of cisplatin or 30 µM cPVP for two hours, then harvested by scraping into ice-cold PBS. DNA was extracted and quantified as mentioned above, taking 2 µg of each DNA sample for library preparation. Damage-seq was performed as previously described by Hu et al. [43] without biotin purification after the primer extension step [44]. Briefly, genomic DNA was sheared by sonication with Bioruptor Pico sonicator (Diagenode Inc., Denville, NJ, USA) to generate fragments averaging 300 bp in length. Damaged DNA immunoprecipitation was performed as described previously, with anti-cisplatin modified DNA antibody (ab103261, Abcam, Cambridge, UK). Library quality was assessed using Agilent 4200 TapeStation. Qualified libraries were pooled and sequenced on a NovaSeq 6000 Illumina sequencer. Reads were processed following the steps mentioned in Hu et al. [26,43]. Reads containing Ad1 adapters were discarded by cutadapt (version 3.5) [45] and were aligned to GRCh38 genome using bowtie1 (version 1.3.1) [46]. Then, Picard MarkDuplicates [47] (version 2.26.10; http://broadinstitute.github.io/picard accessed on 10 August 2023) was used to remove read duplicates. Next, unique reads in BED format were further filtered with Bedtools (version v2.27.1) [48] and custom BASH scripts. To plot average Damage-seq signal at ATAC-seq peaks, the gene’s annotation file was downloaded from Ensembl, assembly GRCh38, release 96. Only peaks that do not overlap genes were obtained using custom scripts and Bedtools (version 2.27.1) slop and merge commands. Reads intersecting peak list were obtained using Bedtools intersect command and profiles flanking ATAC-seq peaks were created using the R (version 4.1.3) Bioconductor genomation package (version 1.26.0) [49]. For box plot analysis, read counts for each peak interval were obtained using Bedtools coverage command, and plotted using ggplot2 package (version 3.3.5 [50]). GG coordinates in the entire human genome were extracted with FUZZNUC (version 6.6.0.0) from the EMBOSS package [51]. GG profiles were plotted in the same way as described for Damage-seq profiles.

### 2.8. Western Blot

A2780 cells were seeded at density of 1.5–3 × 10^6^ cells/well in 6-well plates, and treated for 24 h with either 5 µM of cPVP or bisPhB, or 30 µM cisplatin. Cells were lysed under ice-cold conditions, using 50 mM Tris-HCl (pH 7.5), 150 mM NaCl, 1 mM EDTA, 1% Triton X-100 lysis buffer supplemented with protease inhibitors (539134, Calbiochem, San Diego, CA, USA), and collected by scraping. Lysates were centrifuged at 16,000 rcf, for 10 min at 4 °C, and protein content was quantified utilizing the Bradford assay as above. Equal amounts of 45 μg protein samples were electrophoresed using 12% TGX stain-free gel (Bio-Rad, Hercules, CA, USA) in TG-SDS running buffer (Bio-Lab ltd., Jerusalem, Israel) and blotted onto PVDF membranes (Trans-Blot Turbo Transfer Pack; Bio-Rad Hercules, CA, USA). The membrane was blocked using 5% (*w*/*v*) Difco^TM^ skim milk (232100, BD Life Sciences, Spark, MD, USA) in TBST solution (50 mM Tris/Tris-HCl, 150 mM NaCl; Bio-Lab ltd., Jerusalem, Israel. 0.1% Tween^®^ 20 Detergent) for 1 h and incubated over-night with primary anti-H3K9Ac antibody at 4 °C (C5B11, 9694T, Cell Signaling Technology, Danvers, MA, USA). Membranes were incubated for 1 h with secondary anti-rabbit HRP-conjugated antibody (NA934, Cytiva, Marlborough, MA, USA) in 5% (*w*/*v*) Difco^TM^ skim milk and developed using the ChemiDoc XRS+ imager (Bio-Rad, Hercules, CA, USA).

### 2.9. ATAC-Seq

ATAC-seq was performed as previously published [25] using an ATAC-seq kit (53150, Active motif, Carlsbad, CA, USA) and according to the manufacturer’s protocol on 100,000 cells treated with 30 µM of cPVP or 200 µM of cisplatin for two hours. Prior to beginning the ATAC-seq protocol, drug-containing media was replaced with 200 units/mL DNase (LS002006, Worthington Biochemical Corp, Lakewood, NJ, USA) in 1.2 mM MgCl2 and 0.5 mM CaCl2 for 30 min. ATAC-seq libraries were purified and size-selected with HighPrep PCR beads (AC-60005, MagBio, Gaithersburg, MD, USA). Two rounds of size selection were performed. In the first, 0.6× beads were added, and the supernatant recovered. In the second, 0.6× beads were added to the supernatant and DNA was eluted from the beads in 10 mM Tris-HCl. Library quality was assessed using Agilent 4200 TapeStation (Agilent Technologies, Santa Clara, CA, USA). Libraries were pooled and sequenced using an Illumina NextSeq500 sequencer (Illumina, San Diego, CA, USA). Sequencing quality was assessed using FastQC (version 0.11.9; https://qubeshub.org/resources/fastqc accessed on 10 August 2023). The adapter sequence was trimmed from each read using Cutadapt (version 1.15) with the command options: -m 10. Reads were aligned to the genome using Bowtie2 [52] with the command options: –very-sensitive-k 10. For each sample we obtained at least 9 million unique aligned reads. Following alignment, reads that were mapped to chromosome Y or mitochondrial chromosome were filtered and PCR duplicates were removed using Picard MarkDuplicates (version 2.26.10 (http://broadinstitute.github.io/picard accessed on 10 August 2023)). All bam files of replicates from each condition were merged to create a pooled bam file. Peaks were called separately for each replicate and pooled bam files using macs2 callpeak command (version 2.2.7.1) [53]. Peak files from replicates of the same condition were merged using Bedtools intersect to create one set of peaks per condition. Peak regions were chosen only if there was more than 50% overlap of the peak region between all replicates per condition. Differential peak analysis was done using R (version 4.1.3) Bioconductor DiffBind package (version 3.4.11) [54] and plotted using EnhancedVolcano package (version 1.12.0; https://github.com/kevinblighe/EnhancedVolcano accessed on 10 August 2023).

### 2.10. Nrf2 Nuclear Translocation

A2780 cells were seeded at density of 1.5 × 10^6^ cells/well in 6-well plates. Cells were incubated for 4 h with 20 and 70 µM of cisplatin, or 3 and 10 µM of cPVP, followed by trypsinization and centrifugation at 600 rcf for 5 min at 4 °C. Cytosolic and nuclear fractions were then isolated according to the protocol of the Nuclear/Cytosol Fractionation Kit (K266, BioVision, Inc., Mountain View, CA, USA), and protein levels of each fraction were quantified utilizing the Bradford assay [55] with the protein assay dye reagent) Bio-Rad, Hercules, CA, USA) according to manufacturer’s protocol. Proteins from each fraction (5 µg) were loaded on designated 96-well plate of the Nrf2 Transcription Factor Assay Kit (600590, BioVision, Inc., Mountain View, CA, USA) for Nrf2 level determination, according to the manufacturer’s protocol. Nrf2 translocation for each treatment was calculated by the ratio of nuclei-to-cytoplasm Nrf2 levels (nuc/cyto ratio) and was normalized to the same ratio of the control sample (UT). Values higher than 1 indicate Nrf2 translocation to the nucleus relative to control.

### 2.11. Lipid Peroxidation

A2780 cells were seeded in 96-well plates at a density of 25 × 10^3^ cells/well. Cells were incubated for 6 h with treatments ranging from 0 to 6 µM of cPVP or 0–40 µM of cisplatin, before the ROS generating agent 2,2′-Azobis(2-amidinopropane) dihydrochloride (AAPH; final conc. 1 mM in media; 08963, Polysciences, Inc., Warrington, PA, USA) was added for over-night incubation. Lipid peroxidation levels were evaluated by adjusted thiobarbituric acid reactive substances (TBARS) assay [56]. From each well, 50 µL of culture media was transferred to 96-well plate, followed by addition of 150 µL 0.57% TBA (Sigma-Aldrich, Rehovot, Israel) in 30% glacial acetic acid (J.T. Baker, Phillipsburg, NJ, USA). Plate was sealed and incubated at 95 °C for 1 h before being removed to ice for 10 min until reaching room temperature. Absorbance at λ = 532 nm was immediately measured by the Cytation 3 Imaging Reader. In parallel, MTT assay was conducted on treated cells as described above. Final results were obtained by normalizing TBAR OD to MTT OD for each well.

### 2.12. XR-Seq

A2780 cells were treated for 3 h with either 30 µM of cPVP or 200 µM of cisplatin. XR-seq was performed as previously described [25,26]. Briefly, 390 × 10^6^ cells per library were harvested and collected by centrifugation. Cells were resuspended in lysis buffer and lysed using syringes sequentially with 25 and 27 G needles. Low-molecular-weight DNA was isolated, and samples were incubated with RNase-A (1007885, Qiagen, Hilden, Germany). Primary excision products were pulled down by TFIIH co-immunoprecipitation using anti-p62 and anti-p89 antibodies (sc-293 and sc-292, Santa-Cruz Biotechnology, Dallas, TX, USA). Adapters were ligated on both ends of the excised oligos and immunoprecipitated by the anti-cisplatin antibody. The damages induced by cisplatin were reversed in vitro by incubating the samples with 200 mM NaCN at 65 °C over-night. DNA was PCR-amplified and purified by native polyacrylamide gel electrophoresis (PAGE). Library quality was assessed using Agilent 4200 TapeStation. Libraries were pooled and sequenced on a HiSeq 2500 Illumina sequencer (Illumina, San Diego, CA, USA). Quality score for each nucleotide was analyzed using the fastx-toolkit to ensure only high-quality reads were processed. The adapter sequence was trimmed from each read using Trimmomatic [57] version 0.36 with the command options: ILLUMINACLIP:adapter_sequence.txt: 2:30:10; using this adapter sequence: TGGAATTCTCGGGTGCCAAGGAACTCCAGTCACNNNNNNATCTCGTATGCCGTCTTCTGCTTG. Following adapter removal, 50 nt length reads were filtered. Reads were aligned to the genome using Bowtie (version 1.3.1) with the command options: -q –nomaqround –phred33- quals -p 32 -m 4 -n 2 -e 70 -l 20 –chunkmbs 800 –best –S. For each sample we obtained 4.6–7.8 million unique aligned reads. Following alignment, reads mapped to chromosome Y or mitochondrial chromosome were filtered and PCR duplicates were removed using Picard MarkDuplicates (version 2.26.10 (http://broadinstitute.github.io/picard accessed on 10 August 2023). To plot average XR-seq signal along genes, the gene’s annotation file was downloaded from Ensembl, assembly GRCh38, release 96. Non-overlapping regions around the TSS were obtained using custom scripts and Bedtools (version 2.27.1) slop and merge commands. Only non-overlapping genes that were longer than 5000 were included in the plot. Reads intersecting gene list were obtained using Bedtools intersect command and strand-specific profiles over the TSS were created using the R (version 4.1.3) Bioconductor genomation package (version 1.26.0). For box plot analysis, read counts for each gene interval were obtained using Bedtools coverage command, and plotted using ggplot2 package (version 3.3.5). Normalized profiles of XR-seq at ATAC-seq peaks were created as described for Damage-seq profiles. All XR-seq profiles were normalized to Damage-seq profiles.

### 2.13. Resistance Induction

The long-term exposure protocol was based on the work of Behrens et al. [58]. Freshly thawed A2780 cells were given one week of recovery before determining initial IC_50_ values for cisplatin and cPVP using the MTT assay, as described above. Cells were cultured under three conditions: long-term cPVP exposure, long-term cisplatin exposure, and unexposed control. Initial drug concentrations were 0.003 × of the initial IC_50_ (0.02 nM of cPVP and 4 nM of cisplatin), introduced to cells three times for 72 h periods over 3 weeks, allowing growth to recover between cycles. Following 3 cycles of exposure, drugs doses were doubled and procedure was repeated until exposure dosage reached 4 times the initial IC_50_ values. With high doses, cycles were extended to 2 weeks each, in order to allow growth recovery before the next exposure. After 36 cycles of exposure, cells were cultured in drug-free media for an additional 4 weeks, and final IC_50_ values were assessed by the MTT assay.

### 2.14. Statistical Analysis

All data represents the results from at least two biological replicates performed in at least two technical repetitions. Values are reported as mean ± SEM unless otherwise specified. Statistical significance was calculated by Student’s *t*-test for cisplatin vs. cPVP unless otherwise mentioned. Significance is represented by * *p* < 0.05, ** *p* < 0.01, and *** *p* < 0.001.

## 3. Results

### 3.1. Synthesis of a Novel Triple-Action Prodrug Composed from Cisplatin, PhB and VPA

**Figure 2:**
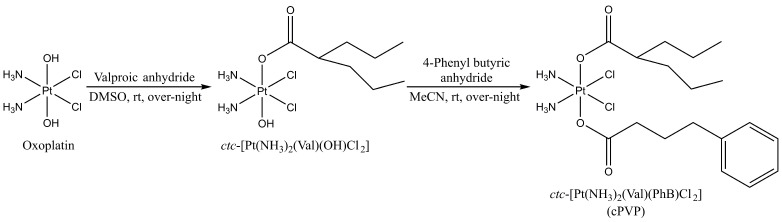
Schematic representation of cPVP synthesis. Cisplatin was oxidized by H_2_O_2_ to oxoplatin, which was successfully carboxylated on axial positions with the anhydrides of VPA and PhB, in a stepwise manner. rt—room temperature.

The prodrug synthesis started from oxoplatin {*ctc*-[Pt(NH_3_)_2_(OH)_2_Cl_2_], the Pt(IV) derivative of cisplatin with two OH axial ligands}. The two hydroxido groups present in the axial positions were functionalized via esterification by the anhydride of the corresponding bioactive ligands, in a stepwise manner (Figure 2). In brief, oxoplatin was reacted over-night with ~1.5 equivalents of valproic anhydride in DMSO to yield the intermediate product monovalproato-monohydroxido-oxoplatin, *ctc*-[Pt(NH_3_)_2_(VPA)(OH)Cl_2_], which was reacted further with five equivalents of 4-phenylbutyric anhydride to yield the desired biscarboxylato product, *ctc*-[Pt(NH_3_)_2_(VPA)(PhB)Cl_2_] (cPVP). The progress of the reactions was monitored by ^195^Pt NMR, and the HPLC-purified final product was characterized by ^1^H, ^13^C and ^195^Pt NMR spectroscopy, as well as by ESI-MS and elemental analysis (Supplemental Figure S1A–E). cPVP was stable after storage at −20 °C for at least 3 years (Supplemental Figure S1F).

### 3.2. cPVP Is a More Efficient Genotoxic Agent than Cisplatin

**Figure 3:**
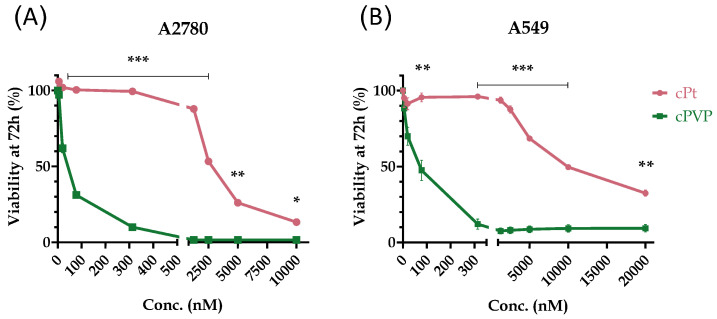
Cytotoxicity of cPVP and cisplatin (cPt). (**A**) A2780 cells were incubated for 72 h with ascending concentrations of the drugs (0–10,000 nM), followed by MTT assay. (**B**) Same as A except data is presented for A549 cells with drug concentrations ranging 0–20,000 nM; data presented as percent of viability normalized to untreated control. Results are based on at least two biological replicates performed in quadruplets. Graphs present mean ± SEM. * *p* < 0.05; ** *p* < 0.01; *** *p* ≤ 0.001 based on Student’s *t*-test.

In order to evaluate the efficacy of cPVP as an anti-cancer agent, we compared its cytotoxic effect to that of cisplatin. A2780 ovarian cancer and A549 lung cancer cell lines were treated with ascending concentrations of cPVP or cisplatin for 72 h, followed by an MTT cell-viability assay and IC_50_ assessment. Previously published IC_50_ values for cisplatin are 0.5–1.1 µM for A2780 cells [58,59] and 3.8–5.95 µM for A549 cells [59,60]. For both cell lines, cPVP was much more toxic than cisplatin (Figure 3A,B), with up to three orders of magnitude lower IC_50_ values (Table 1). As a control, we also compared the cytotoxic effect of cPVP to the co-administration of its components. cPVP was found to be more toxic, as the co-administration of its components presented an identical cytotoxic profile to that of cisplatin alone (Supplemental Figure S2, Table 1).

### 3.3. Inhibition of KDACs by cPVP Increases Chromatin Accessibility and Inhibits Cell Defenses

**Figure 4:**
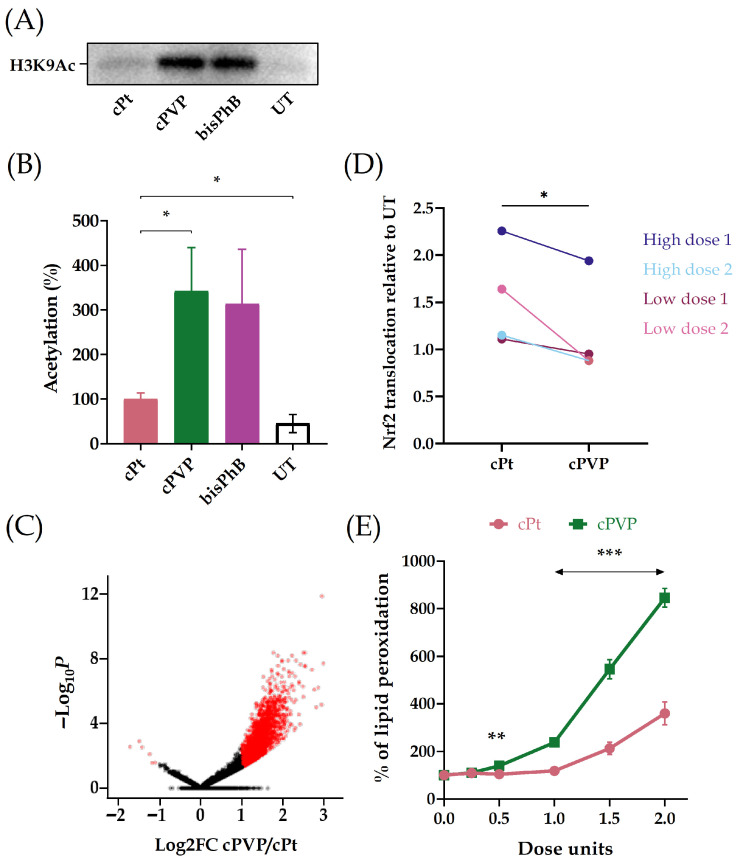
The PhB and VPA components of cPVP inhibit KDAC activity. (**A**) Elevated levels of histone H3 acetylation in the presence of cPVP. A2780 cells were treated with 5 µM of cPVP, 5 µM of bisPhB or 30 µM of cPt for 24 h. Shown is a representative Western blot for H3K9Ac. (**B**) Quantification of Western blots from three biological replicates. Acetylation under cPt treatment was defined as 100%. Band intensity was normalized to total protein. Mean ± SEM. * *p* < 0.05, for one-tailed Student’s *t*-test. (**C**) Volcano plot for differential chromatin accessibility at ATAC peaks in cPVP vs. cPt treated samples. A2780 cells were treated with either 30 µM of cPVP or 200 µM of cPt for two hours prior to ATAC-seq. Differentially accessible regions are depicted in red. (**D**) cPVP reduces Nrf2 translocation to the nucleus relative to cisplatin. A2780 cells were incubated for four hours with either 3 µM of cPVP vs. 20 µM of cPt, or 10 µM of cPVP vs. 70 µM of cPt. Nucleic and cytosolic fractions were extracted and Nrf2 levels were quantified in each. Data expressed as the ratio between treated and untreated nuclei-to-cytoplasm Nrf2 level ratios. Results are based on two biological replicates for each set of treatments. * *p* < 0.05 for paired Student’s *t*-test. (**E**) cPVP decreases cells’ ability to defend against oxidative stress. A2780 cells were treated with a range of 0–2 doses of cPt or cPVP for six hours, followed by over-night ROS induction and TBAR assay. Lipid peroxidation levels were normalized to cell viability using MTT assay. Data presented as percent of untreated control. One dose unit was defined as 20 µM for cPt and 3 µM for cPVP. Results are based on four biological replicates performed in quadruplets. Mean ± SEM. ** *p* < 0.01, *** *p* ≤ 0.001 for Student’s *t*-test.

We validated the effect of cPVP on KDAC activity by assessing the acetylation levels of histone H3. A2780 cells were cultured for 24 h with cisplatin, cPVP or bisPhB (*cct*-[Pt(NH_3_)_2_(PhB)_2_Cl_2_]), which is a previously reported Pt(IV) prodrug known to inhibit KDAC activity [39], and served as positive control. Substantially higher acetylation levels of histone H3 were found in the presence of cPVP or bisPhB vs. cisplatin treatment (Figure 4A,B; Supplemental Figures S3 and S4). Since KDAC activity regulates chromatin condensation, we employed ATAC-seq to evaluate the effect of cPVP on chromatin accessibility across the genome [61]. cPVP treatment resulted in elevated accessibility compared to cisplatin treatment (Figure 4C), consistent with KDAC inhibition.

The VPA component of cPVP has also been reported to inhibit nuclear factor erythroid 2-related factor 2 (Nrf2) [62,63,64], which is responsible for the antioxidant and detoxifying defense system of the cell [65]. In order to evaluate the inhibitory effect of cPVP on the Nrf2/Keap1 pathway, we examined its influence on Nrf2 translocation into the nucleus, a crucial step in the pathway’s activation. A2780 cells were incubated with sets of either low or high doses of cPVP and cisplatin for four hours. Next, cytosolic and nuclear fractions were extracted from cells (Supplemental Figure S5) and were used for Nrf2 sub-cellular partition quantification. At similar effective doses, cPVP reduces the translocation of Nrf2 into the nucleus relative to cisplatin (Figure 4D).

Since Nrf2 activation enhances the antioxidant capacity of the cell, we investigated whether the diminished translocation of Nrf2 to the nucleus following cPVP treatment is reflected in the ability of the cells to defend against oxidative stress. A2780 cells were treated with cisplatin or cPVP for six hours followed by the addition of an oxidative stressor for over-night incubation. The levels of lipid peroxidation products were then measured and normalized to cell viability using the TBAR and MTT assays, respectively. At similar effective doses, cPVP treatment led to a significant increase in lipid peroxidation levels compared to cisplatin treatment (Figure 4E), suggesting that cPVP impedes the cells’ protective response against oxidative stress. These results provide further support for Nrf2 inhibition by cPVP.

### 3.4. cPVP Increases DNA Damage Formation

**Figure 5:**
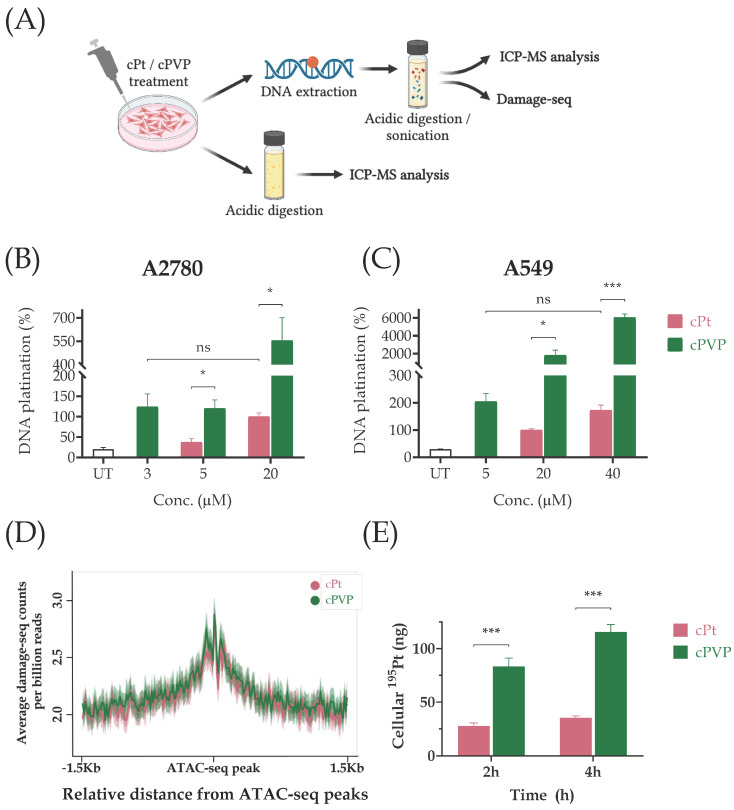
cPVP induces higher damage formation independent of its effect on chromatin accessibility. (**A**) Outline of sample preparation for DNA damage assessment, Damage-seq, and cellular uptake evaluation. (**B**) DNA damage formation following cPt and cPVP treatments in A2780 cells treated with cPt or cPVP. Cells were incubated with treatments for two hours before DNA was extracted and subsequently subjected to ICP-MS analysis of ^195^Pt content. The damage caused by 20 µM of cPt was defined as 100% DNA platination. Results are based on two biological replicates performed in duplicate. (**C**) Same as B except data presented for A549 cells. (**D**) Average Damage-seq signal following cPt and cPVP treatments in accessible regions in A2780 cells. Damage signal is plotted at the 3 Kb regions flanking ATAC-seq peaks from cells treated with either cPt or cPVP. Shadow represents 95% confidence interval for the mean. (**E**) Cellular uptake of cPt (20 µM) and cPVP (3 µM) after two and four hours of incubation in A2780 cells. Treated cells were digested by nitric acid for subsequent ^195^Pt content determination by ICP-MS. Absolute given doses of ^195^Pt: 3900 ng from 20 µM of cPt, and 580 ng from 3µM of cPVP. Data presented as absolute ^195^Pt amounts detected within cells. Results are based on four biological replicates performed in duplicate. All bar graphs indicate mean ± SEM. * *p* < 0.05; *** *p* ≤ 0.001; ns—not significant for Student’s *t*-test.

Cisplatin cytotoxicity is mainly attributed to its DNA damaging property [12,13]. We assessed the ability of cPVP to do the same. A2780 and A549 cells were exposed to varying doses of cPVP or cisplatin for two hours followed by DNA extraction. The platinum content in DNA samples was measured by Inductively Coupled Plasma Mass Spectrometry (ICP-MS; Figure 5A). Consistent with the higher cytotoxicity, cPVP caused significantly more DNA damage than cisplatin at each concentration (Figure 5B,C).

cPVP requires much lower doses than those of cisplatin, both in terms of cytotoxicity and of damage induction. For our subsequent experiments, we sought to treat cells with similar effective doses. Based on DNA damage levels, for the A2780 cell line, sub-lethal doses that elicit damage would undergo a short (two-hour) incubation with 3 µM cPVP and 20 µM cisplatin, and for A549 cells, 5 µM cPVP vs. 40 µM cisplatin (Figure 5B,C). To facilitate accurate comparisons between treatments our follow-up experiments were performed using these ratios as guidelines to obtain similar effective doses.

KDAC inhibition elevates chromatin acetylation and can subsequently increase chromatin accessibility. Thus, KDAC inhibitors could sensitize the genome to damage. Based on genome-wide mapping of cisplatin adducts, however, adduct formation is not strongly affected by the chromatin structure [26]. To test if the higher damage levels after cPVP treatment reflect enhanced damage formation at accessible chromatin, we mapped cisplatin adducts by Damage-seq in A2780 cells treated with either cisplatin or cPVP for two hours. Accessible regions were determined by ATAC-seq performed under the same conditions (methods and [25]). The higher cisplatin adduct frequency in these accessible regions reflects the elevated frequency of GG di-nucleotides, the main target for cisplatin adducts (Supplemental Figure S6). Treatment with cPVP did not alter the damage profiles, which were almost identical for both treatments (Figure 5D).

The higher DNA damage induced by cPVP could be due to efficient cellular uptake of the prodrug. To test this, A2780 cells were incubated with drug concentrations that induce similar damage levels (3 µM for cPVP and 20 µM for cisplatin) for two and four hours, and the cellular platinum content was measured using ICP-MS. At each time point, the cellular accumulation of cPVP was greater than that of cisplatin (Figure 5E). The ^195^Pt content after cPVP treatment revealed a significantly higher platinum content associated with the cells compared to cisplatin treatment, despite the lower concentration of cPVP. Moreover, cisplatin exhibited minimal cellular accumulation even after four hours (approximately 1%), while cPVP cellular uptake reached 20% of the initial dose. Thus, the increased cell-associated platinum concentration indicates there is increased cellular uptake of cPVP and supports its enhanced ability to cause DNA damage.

### 3.5. cPVP Inhibits Nucleotide Excision Repair in Cancer Cells

**Figure 6:**
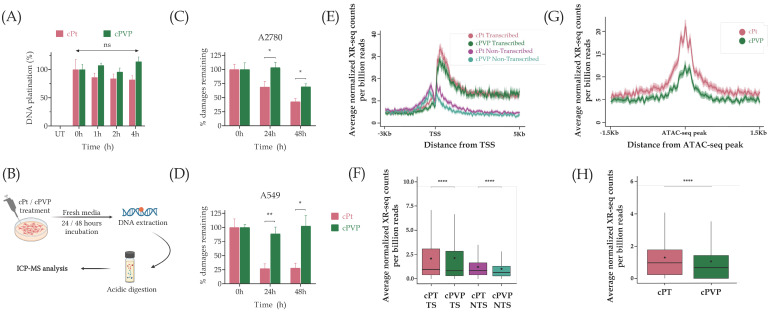
cPVP inhibits nucleotide excision repair in cancer cells. (**A**) DNA damage accumulation post-treatment. A2780 cells were treated with 3 µM of cPVP or 20 µM of cPt for two hours prior to treatment removal. DNA was extracted after 0, 1, 2 and 4 h post-treatments and subjected to ^195^Pt content determination by ICP-MS. The initial damage measured at time of wash (0 h) was defined as 100% DNA platination for each drug. Results are based on four biological replicates performed in duplicate. Mean ± SEM. ns—not significant for Student’s *t*-test compared to initial damage from each drug. (**B**) Outline of DNA repair kinetics assay. (**C**) DNA repair kinetics following cPt and cPVP treatments in A2780 cells treated with 3 µM of cPVP or 20 µM of cPt. Drug-containing media was replaced after two hours of incubation and DNA was extracted at 0, 24 and 48 h post-treatment for ICP-MS analysis. The initial damage was defined as 100% for each drug. Results are based on two biological replicates performed in duplicate. Mean ± SEM. * *p* < 0.05, for Student’s *t*-test. (**D**) Same as C except A549 cells were treated with 5 µM of cPVP or 40 µM of cPt. * *p* < 0.05, ** *p* < 0.01 for Student’s *t*-test. (**E**) Average XR-seq repair signal normalized to Damage-seq signal over annotated genes plotted 3 Kb upstream and 5 Kb downstream of the transcription start site (TSS, *n* = 6597). Signal is plotted separately for the transcribed and non-transcribed strands. A2780 cells were treated with 30 µM of cPVP or 200 µM of cPt for two hours prior to XR-seq libraries preparation. The data represent the average of two biological replicates. Shadow represents 95% confidence interval for the mean. (**F**) Total XR-seq read counts over the same genes as E in the transcribed and non-transcribed strands. **** *p* < 0.0001, based on Wilcoxon signed-rank test with Bonferroni correction. Boxes represent range between 75th and 25th percentile, the line represents the median and the diamond the mean. (**G**) Average XR-seq repair signal normalized to Damage-seq signal at the 3 Kb regions flanking ATAC-seq peaks representing accessible chromatin regions. Average cPt and cPVP repair signals are plotted for 7526 and 12,048 ATAC-seq peaks measured in cPt and cPVP treated cells, respectively. A2780 cells were treated with 30 µM of cPVP or 200 µM of cPt for two hours prior to ATAC-seq and three hours prior to XR-seq libraries preparation. The data represent the average of two biological replicates. Shadow represents 95% confidence interval for the mean. (**H**) Total XR-seq read counts over the same ATAC-seq peaks as (**G**). **** *p* < 0.0001, based on Wilcoxon signed-rank test with Bonferroni correction. Boxes represent range between 75th and 25th percentile, the line represents the median and the diamond the mean.

To measure repair, we followed the decrease from the initial damage levels over time. Correct measurement of the initial damage levels necessitated the cessation of DNA damage accumulation following the removal of treatments. To validate this, A2780 cells were treated with cisplatin or cPVP for two hours, followed by the removal of treatments. DNA was extracted from cells sampled up to four hours post-treatment, and analyzed for the ^195^Pt content using ICP-MS. There was no noteworthy accumulation or reduction in DNA damage at any of the time points for both treatments (Figure 6A), indicating the damage levels measured after two hours of treatment and drug removal reflect the initial damage levels in repair experiments. To assess the impact of cPVP on DNA repair, A2780 and A549 cells were treated with similar effective doses of cisplatin or cPVP for two hours. Drug-containing media was replaced with fresh media, and cells were sampled at 0, 24, and 48 h. DNA was extracted from the cells and subjected to measurement of the ^195^Pt content by ICP-MS (Figure 6B). In both cell lines, adduct repair in cPVP-treated cells was suppressed compared to cisplatin treatment (Figure 6C,D). In A2780 cells, no repair was observed 24 h after cPVP treatment, contrasting with 30% repair observed after cisplatin treatment. Furthermore, in A549 cells, no significant repair was observed even 48 h following cPVP treatment, as opposed to 70% repair after cisplatin treatment. These results indicate a strong inhibition of NER by cPVP.

To elucidate the inhibitory effect of cPVP on NER we performed XR-seq, a sensitive method for high-resolution genome-wide mapping of the excised oligos released during NER [26,66]. The genome-wide profile of repair was very similar for both damaging treatments. Consistent with our previous cisplatin repair maps, higher repair was measured on the transcribed vs. the non-transcribed strands in genes, indicating a preference for transcription-coupled repair of the cisplatin adducts [25,26]. (Figure 6E,F). Still, a slightly but significantly lower XR-seq signal was observed in cPVP treatment compared to cisplatin alone. Similarly, analysis of repair at accessible chromatin regions (defined by ATAC-seq peaks measured under the same conditions) showed lower cisplatin repair after cPVP treatment (Figure 6G,H). While the overall pattern of repair was similar, the lower enrichment could reflect a lower efficiency of repair in cPVP treatment compared to cisplatin.

### 3.6. cPVP Prevent Resistance in a Cell Line Model

**Figure 7:**
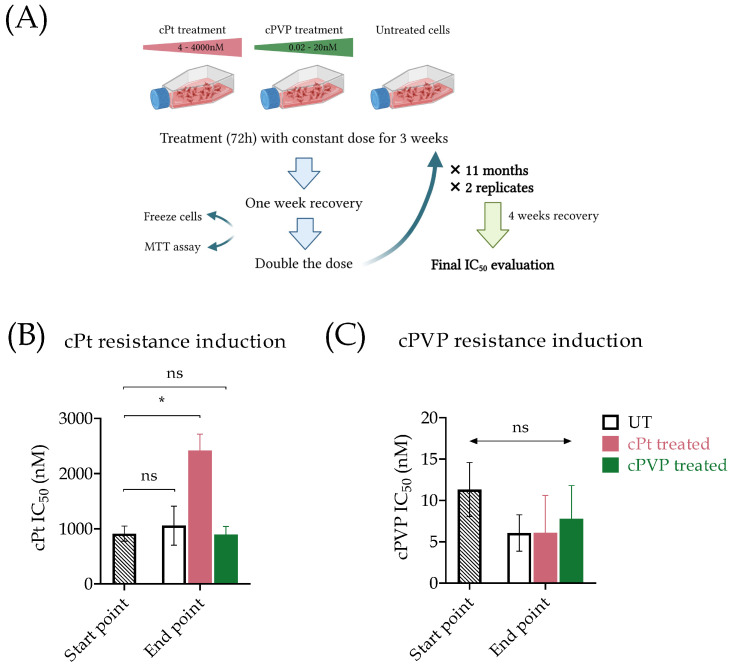
cPVP as a potential anti-cancer drug. (**A**) Outline of prolonged resistance induction assay performed on the A2780 cell line. (**B**) IC_50_ values of cisplatin in A2780 cells, before and after long-term exposure to cPt (4–4000 nM) or cPVP (0.02–20 nM). Results are based on two biological replicates. (**C**) IC_50_ values of cPVP in A2780 cells, before and after long-term exposure to cPt (4–4000 nM) or cPVP (0.02–20 nM). Results are based on two biological replicates. In all graphs, mean ± SEM are presented. * *p* < 0.05; or ns—not significant based on Student’s *t*-test.

We assessed the potential of long-term treatment with cPVP to develop resistance. To induce cisplatin or cPVP resistance in A2780 cells, we exposed them to increasing concentrations of the treatments (Figure 7A). Initial doses were set to 0.003 × IC_50_ of either cPVP or cisplatin. After three weeks of exposure, the doses were doubled. Exposures continued up to doses of 4 × IC_50_, requiring almost a year, followed by a four-week period of recovery in which cells were cultured without any treatment. After this long-term treatment with cisplatin, cells exhibited a significant increase in the cisplatin IC_50_ value, indicating the development of resistance to the drug. In contrast, cells treated with cPVP presented no change in the cisplatin IC_50_ value, indicating that cPVP prevents cells from developing resistance to cisplatin (Figure 7B). Moreover, under both conditions there was no change in cPVP IC_50_ values, suggesting that cPVP not only overcomes cisplatin resistance but also prevents the development of self-resistance (Figure 7C).

## 4. Discussion

Although broadly used, cisplatin-based chemotherapy is limited due to its severe side effects and multifactorial drug resistance [8,9,11]. While the unfavorable toxicity profile of cisplatin (primarily nephrotoxicity and neuropathy) was improved by the development of second-generation platinum agents such as carboplatin [67,68,69], overcoming drug resistance was achieved only for colorectal cancers by the development of oxaliplatin, the third-generation platinum drug [9,69,70,71]. Yet, the need remains for further platinum drugs that circumvent resistance.

The synthesis of Pt(IV) prodrugs is a well-established approach aimed at improving cisplatin treatment, reducing side effects, and potentially overcoming drug resistance [33,34]. Herein, we have utilized this method to synthesize a novel triple-component Pt(IV) prodrug, cPVP, composed from cisplatin, and two KDAC*i*: PhB and VPA. Compared to cisplatin, cPVP demonstrated enhanced efficacy against both a cisplatin-sensitive and -resistant cell line, as indicated by significantly lower IC_50_ values at the nM scale (as opposed to the µM scale for cisplatin), and high DNA-damaging abilities. These findings suggest that cPVP holds promise as an improved treatment option with lower dosage requirements, and could be potentially effective in treating cancers that are resistant to cisplatin. As for side effects, considering the inertness of Pt(IV) derivatives in terms of binding to proteins and nucleophiles in the blood stream [36], it is possible that cPVP will exert fewer side effects than cisplatin. This will be tested in future studies.

The increased cellular uptake of cPVP, attributed to its greater lipophilicity, only partially accounts for its heightened efficiency. While cPVP kills cells 80-180 times more efficiently than cisplatin, it only induces 3–30-fold more DNA damage. Our results indicate that the PhB and VPA components of the drug functionally contribute to this enhanced efficiency. Since PhB and VPA are negatively charged in physiological fluids, they do not penetrate the negatively charged cell membranes resulting in in vitro IC_50_ values in the mM range [38,72]. The conjugation of PhB and VPA to Pt(IV) neutralizes the negative charge of these ligands and increases the lipophilicity compared to cisplatin, resulting in efficient cellular accumulation, significantly decreasing the IC_50_ of cPVP to the nM range. Regarding KDAC activity, the high acetylation of histone H3 and elevated chromatin accessibility measured by ATAC-seq provide evidence for the inhibitory effect of the cPVP components on KDACs (Figure 4A–C).

Cisplatin treatment activates the nuclear factor erythroid 2-related factor 2/Kelch-like erythroid cell-derived protein with the CNC homology-associated protein 1 (Nrf2/Keap1) pathway [73,74], which is responsible for the antioxidant and detoxifying defense system of the cell [65]. Resistant cells exploit Nrf2 activation to enhance their survival and eliminate cisplatin [75,76]. As a prodrug of the Nrf2-inducer cisplatin, and the Nrf2-inhibitor VPA, cPVP possesses the potential for two counter-effects on the Nrf2/Keap1 pathway. Our results indicate that the inhibitory effect of VPA on Nrf2 is stronger than the inductive effect of the cisplatin component. While cisplatin induces Nrf2 translocation to the nucleus, this translocation was reduced after cPVP exposure (Figure 4D). Similarly, the ability of the cells to defend against oxidative stress was significantly diminished under cPVP treatment (Figure 4E).

We previously reported that the chromatin structure affects cisplatin repair but not DNA adduct formation [26]. While cPVP induced higher damage levels, the genome-wide patterns of damage were unaltered. Furthermore, nucleotide excision repair of these adducts was significantly reduced after cPVP treatment, despite the elevated chromatin accessibility that should enhance repair. Together, these findings suggest that the contribution of VPA or PhB (or both) to anti-resistance treatment is not directly mediated by the enhanced chromatin accessibility affecting damage sensitivity. KDACs also deacetylate non-histone proteins, including NER factors [77]. Thus, the inhibition of repair could be a result of the enhanced acetylation of the repair factors themselves [78]. Furthermore, KDAC*i* treatment can lead to the reprogramming of gene transcription, indirectly altering the expression of repair proteins. For these reasons, the KDAC*i* properties of cPVP grant it a wider pharmacological potential that could be assessed in future acetylome and transcriptome studies.

While the most abundant DNA damage induced by cisplatin are intrastrand adducts that crosslink two adjacent purines (primarily Gs), cisplatin also induces interstrand crosslinks, and protein–DNA crosslinks, as well as damage to RNA and proteins [12,13,79]. These could be subject to additional repair mechanisms that were not addressed in this manuscript but could also be affected by the KDAC*i* components.

**Figure 8:**
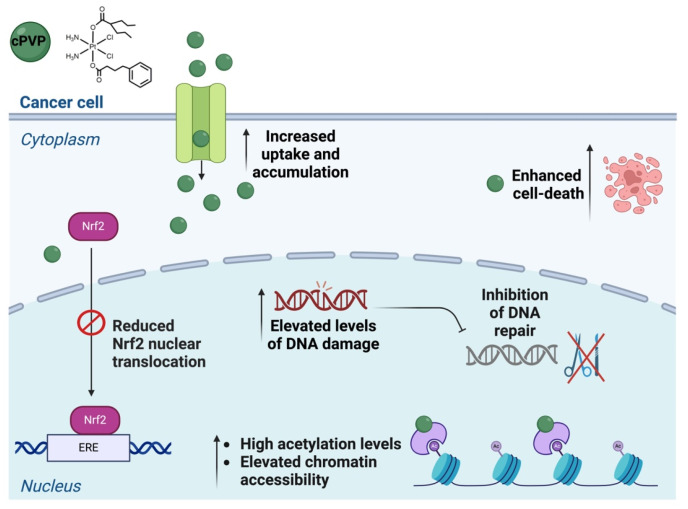
Graph summarizing the multiple simultaneous effects exerted on cancer cells by cPVP.

Though numerous Pt(IV) derivatives have been previously reported, the assessment of their potential to overcome drug resistance was mainly based on IC_50_ values in cisplatin-resistant cell lines [34]. In the case of the novel prodrug cPVP, we conducted a prolonged resistance study that emphasized cPVP’s ability to not only overcome existing cisplatin-resistance, but also to prevent cells from developing resistance to itself or to cisplatin. We attribute these outcomes to the multiple parallel negative effects that cPVP exerts on cancerous cells (Figure 8).

## 5. Conclusions

We have synthesized a novel Pt(IV) prodrug that exerts multiple simultaneous detrimental effects on cancer cells, and appears not to induce resistance. The ease of synthesis, low-dose efficiency, and the fact that cPVP’s sub-components (cisplatin, VPA, and PhB) are all FDA-approved position cPVP as a promising chemotherapy, making it a good candidate for future pre-clinical and clinical studies.

## Figures and Tables

**Table 1:** IC_50_ values of cisplatin, cPVP and co-administration of its components for A2780 and A549 cell lines. Calculated from MTT viability assay after 72 h of incubation with ascending concentrations of either cPt, cPVP, or cPt+PhB+VPA in a 1:1:1 ratio (0–10,000 nM for A2780, and 0–20,000 nM for A549). The IC_50_ values for cisplatin are estimated and resemble previously published values. Based on at least two biological replicates performed in quadruplets. Mean ± SEM.

IC_50_ Value			
	cisplatin (nM)	cPVP (nM)	cPt+PhB+VPA (nM)
A2780	2415.334 ± 52.9	33.0 ± 0.4	2276.4 ± 24.2
A549	10,124.4 ± 1183.7	55.9 ± 17.02	11,600.4 ± 1837.0

## Data Availability

All raw and processed sequencing data generated in this study have been submitted to the NCBI Gene Expression Omnibus (GEO; https://www.ncbi.nlm.nih.gov/geo/) under accession number GSE243355.

## Supplementary Materials

The following supporting information can be downloaded at: https://www.mdpi.com/article/10.3390/biom16040542/s1, Figure S1. Full characterization of cPVP. Figure S2. Co-administration of cisplatin with inhibitors. Figure S3. Full western-blot image corresponding to Figure 4A. Figure S4. Total protein membrane image used to quantify Western blot in Figure 4A. Figure S5. Cytosolic and nuclear fractions analysis. Figure S6. GG profile of A2780 cells.

## Abbreviations

The following abbreviations are used in this manuscript:

AAPH	2,2′-Azobis(2-amidinopropane) dihydrochloride
ATAC-seq	assay for transposase-accessible chromatin using sequencing
BI	biological industries
DMEM	Dulbecco’s modified Eagle’s medium
DMSO	dimethyl sulfoxide
DNA	deoxyribonucleic acid
EDC	1-ethyl-3-(3-dimethylaminopropyl)carbodiimide
EDTA	ethylenediaminetetraacetic acid
ESI-MS	electrospray ionization mass spectrometry
Eq	equivalents
FBS	fetal bovine serum
FDA	food and drug administration
GGR	global genome repair
H3K9Ac	acetylation at the 9th lysine residue of histone H3
HDAC	histone deacetylase
HNO_3_	nitric acid
HPLC	high-performance liquid chromatography
HRP	horseradish peroxidase
IC_50_	half-maximal inhibitory concentration
ICP-MS	inductively coupled plasma mass spectrometry
IV	intravenous
KDAC	lysine deacetylase
KDAC*i*	KDAC inhibitor
Keap1	Kelch-like erythroid cell-derived protein with CNC homology (ECH)-associated protein 1
MTT	3-(4,5-dimethylthiazol-2-yl)-2,5-diphenyltetrazolium bromide
MeOD	deuterated methanol
NER	nucleotide excision repair
NMR	nuclear magnetic resonance
Nrf2	nuclear factor erythroid 2 (NF-E2)-related factor 2
OD	optical density
PAGE	polyacrylamide gel electrophoresis
PBS	phosphate-buffered saline
PCR	polymerase chain reaction
PVDF	polyvinylidene fluoride
PhB	4-phenylbutyric acid
Pt	platinum
RNA	ribonucleic acid
ROS	reactive oxygen species
RP-HPLC	reversed-phase high-performance liquid chromatography
RPMI	Roswell Park Memorial Institute medium
RT	room temperature
SA	Sigma-Aldrich
SDS	sodium dodecyl sulfate
SEM	standard error of the mean
TBA	thiobarbituric acid
TBARS	thiobarbituric acid reactive substances
TBST	Tris-buffered saline with 0.1% Tween^®^ 20 detergent
TCR	transcription-coupled repair
TFIIH	transcription factor II H
TG-SDS	Tris-glycine-SDS buffer
TGX	Tris-Glycine extended
TSS	transcription start site
UT	untreated
UV	ultraviolet
VPA	valproic acid
XR-seq	excision repair sequencing
bisPhB	*cct*-[Pt(NH_3_)_2_(PhB)_2_Cl_2_]
cPVP	*ctc*-[Pt(NH_3_)_2_(VPA)(PhB)Cl_2_]
nt	nucleotides
