# Supplementary material for: A Cisplatin-Based Prodrug Inhibits Nucleotide Excision Repair Independently of Chromatin Accessibility to Overcome Resistance
Source: Biomolecules. 2026 Apr 7;16(4):542. doi: 10.3390/biom16040542 (PMC13113776; doi:10.3390/biom16040542)
Supplement: Supplementary file 1 [file biomolecules-16-00542-s001.zip › biomolecules-4189566-supplementary.pdf]

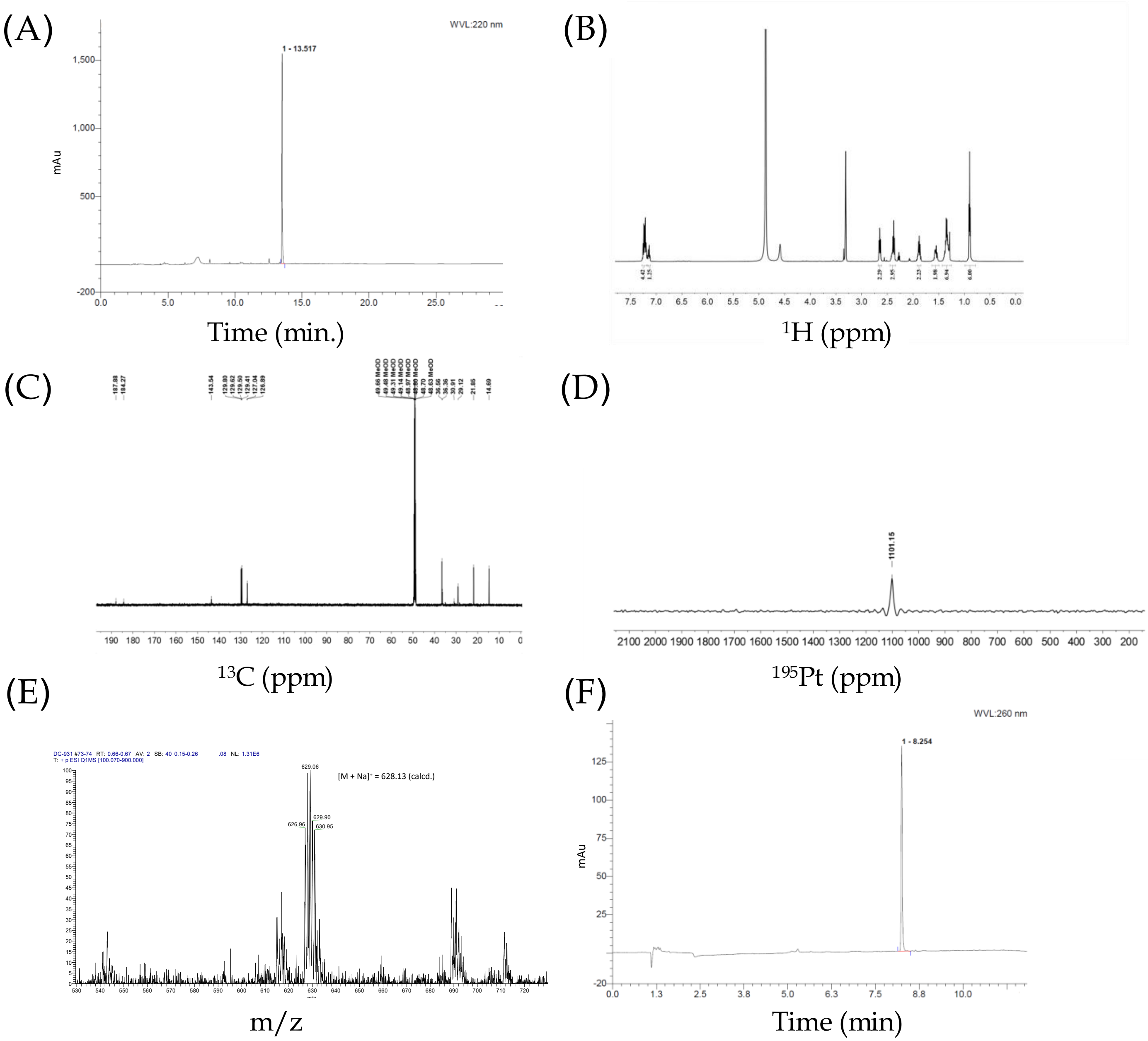

Figure S1. Full characterization of cPVP. (A) HPLC chromatogram of cPVP ran with (0 – 100)% acetonitrile gradient over 15 min followed by 15 min at 100% acetonitrile, in a reverse-phase C18 column (250 mm x 4.60 mm, 5 µm, 100 Å). **(B)** ^{1}H NMR of cPVP in MeOD. **(C)** ^{13}C NMR of cPVP in MeOD. **(D)** ^{195}Pt NMR of cPVP in MeOD. **(E)** ESI-MS (+ve) data of cPVP. **(F)** HPLC chromatogram of cPVP after three years of storage at -20°C. Ran with (0 – 100)% acetonitrile gradient over 25 min followed by 5 min at 100% acetonitrile, in a reverse-phase C18 column (100 mm x 4.60 mm, 2.6 µm, 100 Å).

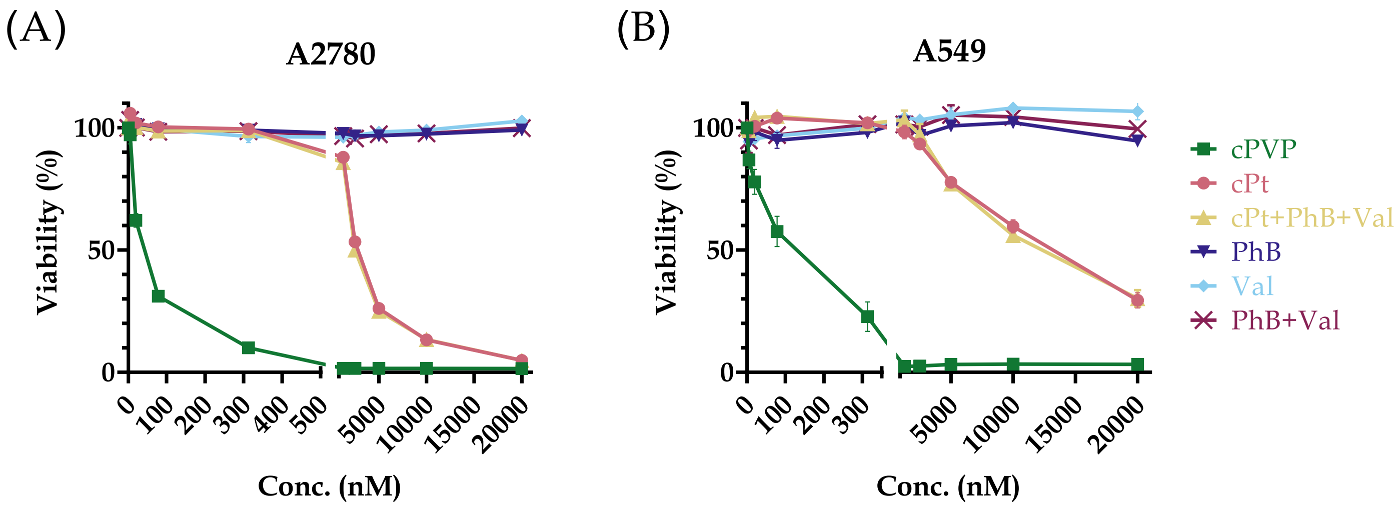

Figure S2. Co-administration of cisplatin with inhibitors. (A) Cytotoxicity of cPt, cPVP and its' components when co-administered in A2780 cells. Cells were incubated for 72 hours with a range of concentrations of either cPVP, cPt, cPt+PhB+VPA (in a 1:1:1 ratio), PhB, VPA, or PhB+VPA (in a 1:1 ratio), followed by MTT assay. Data presented as percent of viability normalized to untreated control. **(B)** Same as A except data presented for A549 cells. Graphs present mean ± SEM. Results are based on at least two biological replicates performed in quadruplets. ****P<0.0001 for all treatments vs. cPVP based on two-way ANOVA for both cell-lines.

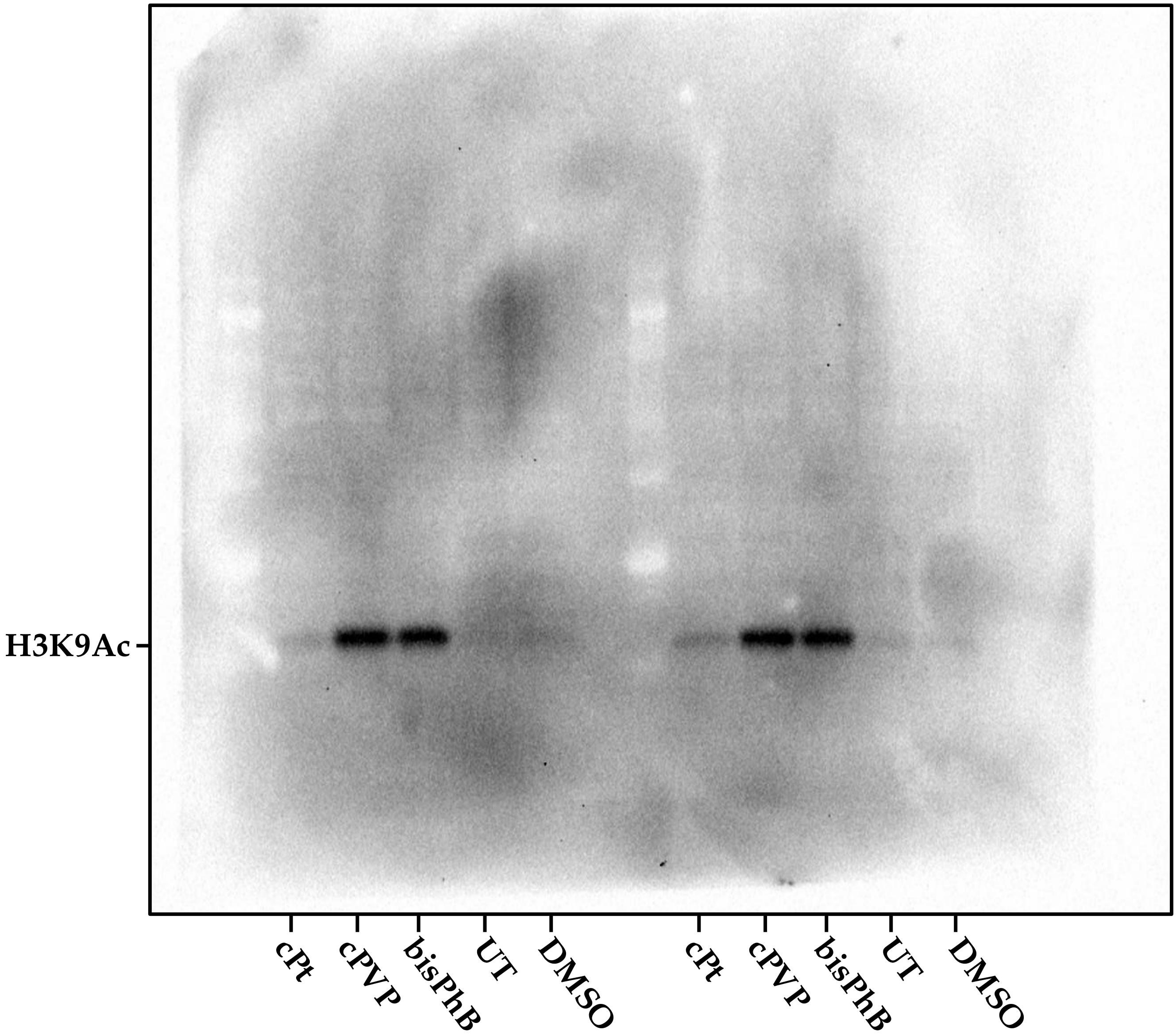

Figure S3. Full western-blot image corresponding to Figure 4A. Histone H3 acetylation analysis using H3K9Ac antibody. A2780 cells were treated with 5 µM of cPVP, 5 µM of bisPhB or 30 µM of cPt for 24 hours.

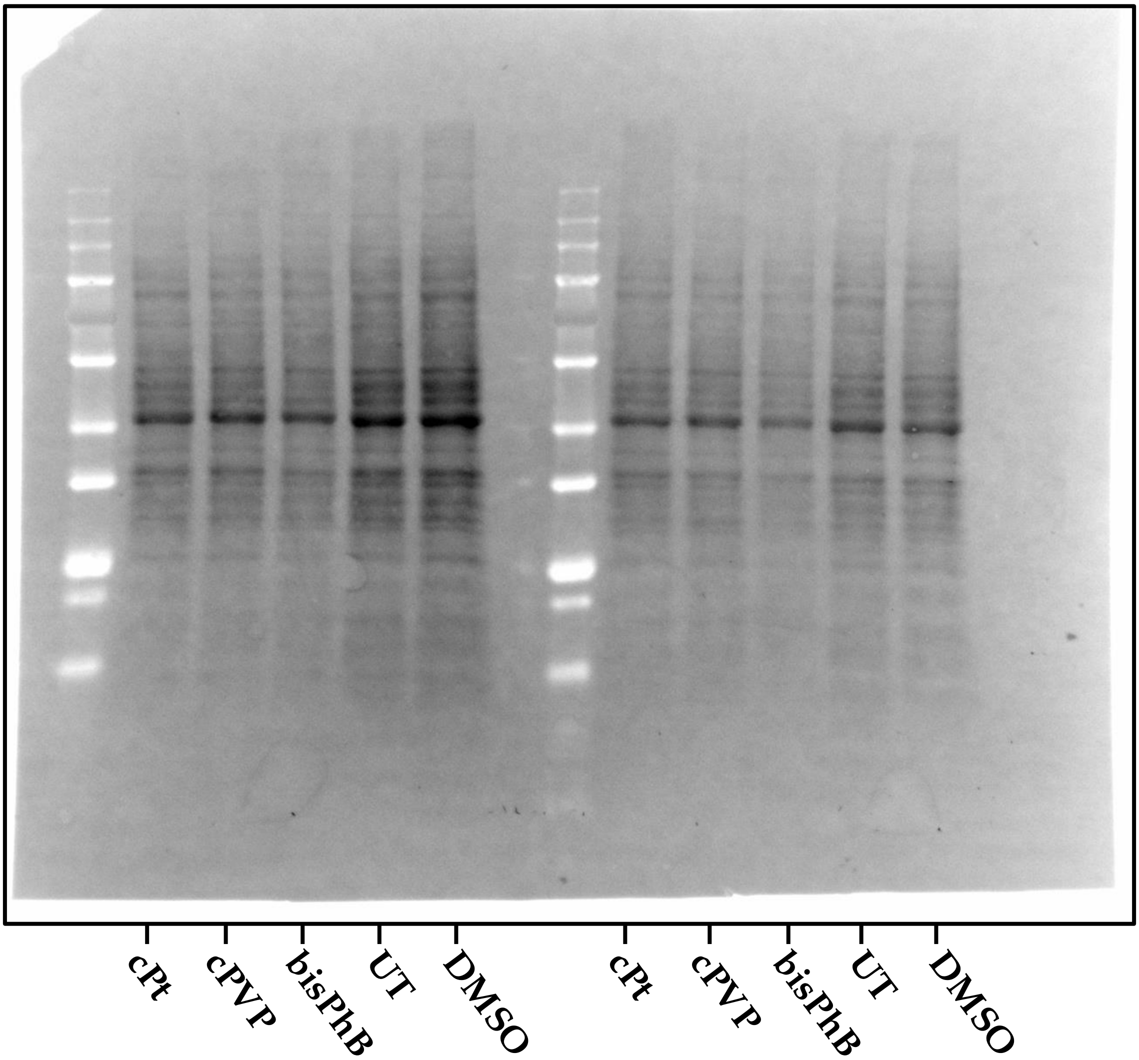

Figure S4. Total protein membrane image used to quantify Western blot in Figure 4A.

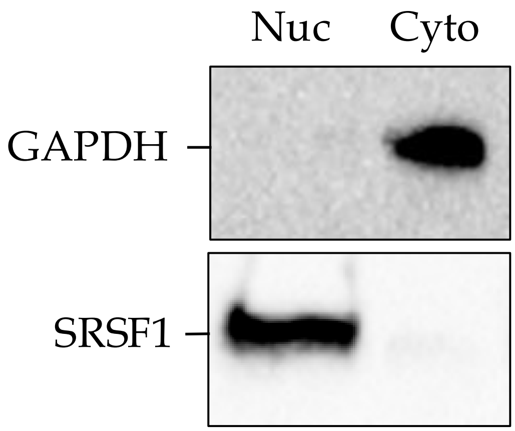

Figure S5. Cytosolic and nuclear fractions analysis. Western-blot for the cytoplasmic marker GAPDH and nucleic marker SRSF1 in nuclear and cytosolic fractions from A2780 cells.

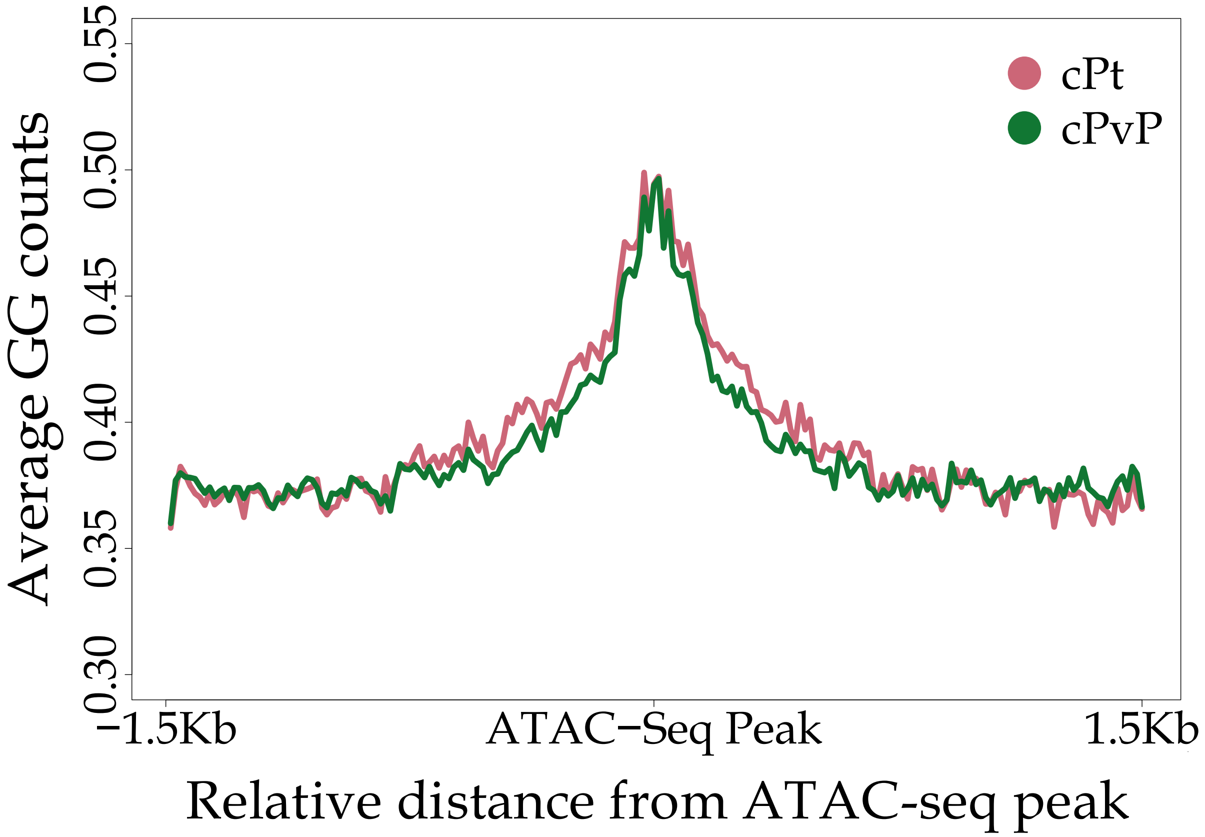

Figure S6. GG profile of A2780 cells. Average GG frequency at ATAC-Seq peaks regions measured following cPt and cPVP treatments in A2780 cells. GG frequency is plotted at ATAC-Seq peaks and 1.5 Kb flanking regions with a bin size of 15 nt. Shadow represent 95% confidence interval for the mean.
